# Prof. Taft’s Criticisms

**Published:** 1876-04

**Authors:** S. P. Cutler

**Affiliations:** Memphis, Tenn.


### PROF. TAFT'S CRITICISMS.

BY S. P. CUTLER, M. D., D. D. S., MEMPHIS, TENN.

In the August and September numbers of the Dental Register will be found Prof. Taft's criticism on a paper of mine, on the subject of amalgam, read before the New York Odontological Society, December, 1874.

I was invited by that society to furnish a paper on some subject of my own choosing, to be read before that society.

Being then engaged on some amalgam experiments, I gave them the results of those experiments with some views and conclusions arrived at concerning those experiments on the subject of amalgam. From the discussions on the paper and several others on the same subject, it would seem that they all met with a favorable reception before that highly intelligent body of leading dentists. This society published those papers under their own sanction unanimously. I did not know what was to be the fate of that paper, as I well knew that there were many who opposed the use of such a material, in that city and other places. If this, and other papers on the subject, met with the almost, if not quite unanimous sanction, it ought to at least, reclaim me from censure, for sending it. In fact I view it in this light—It seems to me that Prof. Taft, in condemning that paper, at the same time condemns also the action of that body on the subject.

If there has, as Prof. Taft says, been an improper use made of this paper by quacks and empirics, I am not alone responsible for it, as I did not publish it, supposing that the society had put that paper under the table or into the fire, what would have been its influence for harm, if harm has been done? I know Prof. Taft is sincere in his opposition to the use of amalgams, still I think he must have very strong prejudices against its use. If Prof. Taft has seen so much evil already from the influence of this paper, as he says he has, I think he ought to relate cases, if they have come under his personal observation, such as horrible salivations, holes eaten through the face, jaw bones all necrosed, corroded throat and stomach, hair dropped from head, tongue scuttled and riddled through and through, coughs, consumption, and what not coming under his immediate observation, I certainly shall publish any and all such cases coming under my own observation, even bad ulcerations about a tooth from the effects of amalgam, and I think it the duty of every one to do so, in order to get at facts on so important a subject. In this way we may gather up facts that we are now ignorant of. I care nothing about failures of quacks to save teeth with this material, as they are failures with any thing and should be no criterion for any one. Prof. Taft knows very well that there are teeth where it is all but impossible to make reliable gold fillings, owing to want of proper conditions for manipulations, that could be filled with amalgam much more readily and I would say reliably; there are others too far gone to stand the packing of gold, others loose and sore, that could be stopped with the plastic filling, so as to do good service, without jaring or straining weakened walls, such walls as would have to be cut away to fill with gold, and by so cutting away, leave no good support for gold. Such cases back of the cuspids should at least be so saved instead of extracting.

There are other cases again, where the parties can not afford to pay for gold, especially in this region of country, that could pay for something a little cheaper; in such cases it is our duty to save their teeth permanently, and in as reasonable a manner as possible.

In all the cases under consideration, if we do not make an effort to save such teeth we are worse than charlatans, who do try to save, even if they make failures.

And right here, allow me to advise all of you, who are too dignified with an auriferous conceit, to come down a round or two, and do your honest and square duty towards your fellow man, and make yourselves more useful than you have ever done before. It appears to me that there is a little too much blue stocking pride amongst some of our fraternity, to use anything but gold in filling teeth.

I freely admit that gold is more sightly than anything else, as a filling material, and right here turns the whole question:—make amalgam as sightly as gold, and gold would be left out in the cold. When none but hopeless cases are filled with amalgam and very little pains taken in the operations, it certainly is not a fair test; this is often times the case to my certain knowledge.

Prof. Taft quotes high authority to prove that mercury readily oxidises when combined with other substances, as metals, grease and other things, let us see. I very well recollect that when a medical student, (that was many years ago) the I was put at the pestle and mortar to make some mercurial ointment, which was to triturate mercury and stale lard together, after pretty constant labor for two weeks there was scarcely any union with the two, and the quick silver was poured back into the bottle and that was the end of that job. The navy surgeons all coat over their steel instruments to prevent rusting with mercurial ointment.

In galvanic batteries, the zinc is coated over with mercury which remains bright until the zinc is all consumed and no action on the mercury takes place. In amalgam fillings no change takes place in the mercury, from the best evidence I have on the subject, the black or dark color is from the silver, *orgent*, and not from the mercury, and there is nothing injurious in this dark oxide of silver.

Speaking of my experiments with the blow pipe, Prof· Taft says: "These experiments elicit nothing that is not entirely familiar to every dentist who works in metals." This may be true, but I must differ with him in this statement in some cases at least.

Now would it not be the true policy for all the dental colleges to teach their classes all about amalgam and its uses as a filling material, as thoroughly as they do the use of the gold, when and under what conditions it should and should not be used, how to mix it, how judge a good from a bad article. The societies should give clinics, with the same pride and pomp as they do with gold. The books on operative dentistry should give thorough instructions on the subject, show the causes of failures and how to remedy them. Very little has been taught in this direction, in consequence we may expect failures from ignorance on the subject. Right here I unconditionally say, that I believe great good would result to the profession, and to humanity, and countless thousands of teeth saved that are now extracted, if this course should be adopted.

If this course is not adopted our leading men alone will be responsible; from the fact that amalgam is extensively used and will continue to be used as long as dentistry lasts; then why not remedy the evil as far as possible? Opposition to its use will not stop it, the fact has gone forth, and why act foolish any longer about it? This opposition seems to me to be the height of folly, unreasonable. At the American Dental Association, last summer, one gentleman after discussing the subject of amalgam, said, he hoped that the subject would never reach the dignity of a discussion, but said he would leave a society that would debar him from using it, so it would seem in this case at least, privilege was, deemed sacred *Cui bono* for privileges.

In natural history a tumble dady has just as much dignity as the lion of the forest, in its way and place in the scale, and naturalists do not ignore the dady because she rolls her eggs in dirt balls to hatch and nourish them. Some steam doctors ignore the use of mercury altogether in their practice, but occasionally have patients badly salivated, but explain the fact away thusly; they say it is the effects of mercury previously given, that they are running out of their system with their medicine. Mind you, no glass houses, no rocks thrown. I must claim honesty of purpose, in the advocacy of amalgams, as their is nothing to be gained by so doing, and make none for sale, neither have I any interest in its manufacture in any form whatever, I wish to secure my fellow men for his good, nothing more, nothing less; we do not live for self alone, in fact, ought not to.

